# Applying quality indictors in Brazilian hospitaly: tool for improving prophylaxis

**DOI:** 10.1186/1753-6561-5-S6-P149

**Published:** 2011-06-29

**Authors:** C Schmitt, RA Lacerda, MC Padoveze, RNT Turrini

**Affiliations:** 1 Adult health nursing, Sao Paulo University, Sao Paulo, Brazil

## Introduction / objectives

The preferred choice of the best surgical antibiotic prophylaxis practice is usually low despite many published guidelines. This study describes the application of quality indicators for antibiotic prophylaxis in a hospital in Sao Paulo, Brazil.

## Methods

A retrospective study was carried out from November 2009 to March 2010. Medical records from adult inpatients submitted to cardiac, neurological and orthopedic clean surgeries were included. The quality indicators for antibiotic prophylaxis conformity were validated in a previous study. They were composed of six parameters of adequacy as stated by the Hospital Infection Control Committee (HICC) guideline. The conformity index was considered 100% when the antibiotic prophylaxis showed adequacy in all parameters evaluated. We investigated the association between conformity in each parameter and selected population characteristics. Analyses were conducted with 5% significance.

## Results

Medical records from 101 (13.5%) cardiac, 128 (17.1%) neurosurgical, and 519 (69.4%) orthopedic surgeries were evaluated. The general conformity index was 4.9%. The greatest number of surgeries where antibiotic prophylaxis was used without HICC indication occurred among orthopedic procedures (n=293). The total conformity index was 5.8%, 3.1% and 3.0% respectively for orthopedic, neurological and cardiac surgeries. The parameter of administration via was the best achieved; the parameter of duration was the worst. No association was identified between parameter conformity and population characteristics.

## Conclusion

This study reveals a low level of acceptance for HICC guidelines for antibiotic prophylaxis. Quality indicators should be fed back to surgical teams on a regular basis to improve surgical prophylaxis.

## Disclosure of interest

None declared.

